# The Associations Between *Helicobacter pylori* Infection, Serum Vitamin D, and Metabolic Syndrome: A Community-Based Study

**DOI:** 10.1097/MD.0000000000003616

**Published:** 2016-05-06

**Authors:** Li-Wei Chen, Chih-Yi Chien, Chia-Wen Hsieh, Liang-Che Chang, Mei-Huei Huang, Wen-Yuan Huang, Sheng-Fong Kuo, Cheng-Hung Chien, Chih-Lang Lin, Rong-Nan Chien

**Affiliations:** From the Department of Gastroenterology and Hepatology (L-WC, C-HC, C-LL, R-NC), Chang-Gung Memorial Hospital and University, Keelung, Taiwan; Community Medicine Research Center (L-WC, C-YC, CWH, S-FK, C-HC, C-LL, R-NC), Chang-Gung Memorial Hospital and University, Keelung, Taiwan; Department of Laboratory Medicine and Anatomic Pathology (L-CC, M-HH, W-YH), Chang-Gung Memorial Hospital and University, Keelung, Taiwan; and Metabolism and Endocrinology (S-FK), Chang-Gung Memorial Hospital and University, Keelung, Taiwan.

## Abstract

The associations between *Helicobacter pylori* infection, serum vitamin D level, and metabolic syndrome (MS) are controversial. The present community-based study aimed to investigate the effect of *H pylori* infection and serum vitamin D deficiency on MS development.

Individuals from the northeastern region of Taiwan were enrolled in a community-based study from March, 2014 to August, 2015. All participants completed a demographic survey and underwent the urea breath test (UBT) to detect *H pylori* infection as well as blood tests to determine levels of vitamin D, adiponectin, leptin, and high-sensitivity C-reactive protein. The ATP III criteria for MS were used in this study.

A total of 792 men and 1321 women were enrolled. The mean age was 56.4 ± 13.0 years. After adjusting for age and sex, the estimated odds of MS development for a UBT-positive subject were 1.503 (95% confidence interval [CI]: 1.206–1.872, *P* < 0.001) when compared to a UBT-negative subject. For participants with vitamin D deficiency (<20 ng/mL), the odds of MS development were 1.423 (95% CI: 1.029–1.967, *P* = 0.033) when compared to those with sufficient vitamin D level (>30 ng/mL). For participants with both *H pylori* infection and vitamin D deficiency, the odds of MS development were 2.140 (95% CI: 1.348–3.398, *P* = 0.001) when compared to subjects without *H pylori* infection and with sufficient vitamin D levels.

*H pylori* infection and vitamin D deficiency could be predictors of MS. For individuals with both *H pylori* infection and vitamin D deficiency, the odds of MS development were 2.140 when compared to individuals without *H pylori* infection and with sufficient vitamin D levels.

## INTRODUCTION

*Helicobacter pylori* (*H pylori*) infection may cause chronic gastritis, resulting in chronic inflammation and immune responses.^1–3^ The inflammatory reactions, which involve many cytokines, including inflammatory cytokines and adipokines,^4–9^ were reported to be associated with insulin resistance (IR) and metabolic syndrome (MS).^10–14^ The relationship between serum vitamin D and MS development has been a matter of debate.^15–19^ According to an Italian study, patients with *H pylori* infection-related gastritis had lower serum vitamin D concentrations.^20^ Other studies also revealed that vitamin D deficiency could be a predisposing factor for autoimmune gastritis and gastric cancer.^21–24^ We hypothesized that *H pylori* infection and vitamin D deficiency might induce local or systemic inflammatory response via an inflammatory cytokine (high-sensitivity C-reactive protein [HS-CRP]) or adipokines (adiponectin, leptin), leading to the development of IR and MS. Therefore, this community cohort study aimed to analyze the association between *H pylori* infection and serum vitamin D. The influences of *H pylori* infection and serum vitamin D level on MS development were also investigated.

## MATERIALS AND METHODS

This community-based study was performed from March, 2014 to August, 2015 in the northeastern region of Taiwan. The inclusion criteria were age >30 years and absence of pregnancy. Individuals who had received vitamin D supplementation, proton pump inhibitors, *H pylori* eradication therapy, or antibiotics potentially influencing the results of serum vitamin D measurements or *H pylori* detection tests within the 3 previous months were excluded. We also excluded patients with possible *H pylori*-related extra-digestive diseases, such as osteoporosis, obesity, autoimmune thyroiditis, lupus, refractory anemia, and idiopathic thrombocytopenia, because these diseases might interfere with MS analysis. All participants completed a demographic survey and underwent a physical examination, urea breath test (UBT) for detecting *H pylori* infection, and blood tests. The demographic survey assessed the past history of systemic diseases, such as diabetes mellitus (DM), hypertension, hyperlipidemia, hematologic disorders, and autoimmune diseases, medication history, including ongoing vitamin D supplementation, proton pump inhibitor therapy, *H pylori* eradication, and antibiotics received within the 3 previous months, and family history. The physical examination included the measurement of heart rate, blood pressure, body weight, body height, and waist girth (circumference). Body mass index (kg/m^2^) was calculated as weight (kg) divided by squared height (m). Waist girth was measured at the midline between the lowest margin of the subcostal rib and the upper margin of the iliac crest. Blood samples were obtained after an overnight fast, and the following parameters were determined: complete blood cell count, liver and renal biochemistry parameters, lipid profiles, fasting sugar and insulin levels, total vitamin D level, and levels of adiponectin, leptin, and HS-CRP. Blood samples were analyzed within 4 hours after collection to determine complete blood cell counts, biochemical parameters, and antibody titers. The assays for adiponectin and leptin were performed using stored serum samples. The serum samples were stored in tubes at −80 °C following centrifugation (3000 rpm at 4 °C for 30 minutes). The Institutional Review Board of the Chang-Gung Memorial Hospital approved this research (IRB No: 103-3886C). All participants agreed to study conditions and signed the informed consent form before the enrollment in this study.

### Urea Breath Test

^13^C-UBT was performed after an overnight fast using the Proto Pylori kit (Isodiagnostika, Canada) containing 75 mg of ^13^C-urea and additives. Two breath samples obtained within a 30-minute interval were analyzed by gas chromatography/isotope ratio mass spectrometry. Results were expressed as delta over baseline (DOB). A local validation test with a DOB cut-off value of 3.5 yielded a sensitivity of 96% (95% confidence interval [CI]: 93%–99%) and a specificity of 98% (95% CI: 93%–102%) relative to the manufacturer's reference.

### Serum Vitamin D

Serum concentrations of vitamin D (25-hydroxyvitamin D [25(OH)D]) were measured using a radioimmunoassay (Vitamin D total, Roche Diagnostics, Mannheim, German) according to the manufacturer's instructions. The electrochemiluminescence binding assay was performed using Elecsys and Cobas immunoassay analyzers, with the measurement ranges of 3.00 to 70.0 ng/mL and 7.50 to 175 nmol/L. Vitamin D status was defined based on the traditional classification as “deficient” (<20 ng/mL, level 1), “insufficient” (20–30 ng/mL, level 2), and “sufficient” (>30 ng/mL, level 3).^25^

### Adiponectin and Leptin Levels

Levels of adiponectin and leptin were evaluated using commercial kits (Human Total Adiponectin/Acrp30, BioVendor Research and Diagnostic system, Minneapolis, MN; Human Leptin ELISA, Clinical Range, BioVendor Laboratory Medicine, Karasek, Czech Republic) according to the manufacturers’ instructions. The method of analysis was the quantitative sandwich enzyme immunoassay.

### Homeostasis Model Assessment of Insulin Resistance (HOMA-IR)

Since IR is one of the key mechanisms for MS development, we assessed IR using the homeostatic model assessment (HOMA-IR) score.^26^ The HOMA-IR score was calculated by the following formula:

Fasting plasma insulin (mU/L) × fasting plasma glucose (mmol/L)/22.5

A higher HOMA-IR score corresponds to lower insulin sensitivity.^27^

### Metabolic Syndrome

A race-specific waist girth threshold based on the NCEP ATP III criteria^28–30^ was utilized to prevent distortions in MS prevalence. The cut-off values for normal waist circumference in Asian men and women were set to 90 cm (35 inches) and 80 cm (31.5 inches), respectively. MS was defined according to the ATP III criteria as the presence of at least three of the following five traits: visceral (abdominal) obesity, determined on the basis of the Asian waist circumference cut-offs (men: >90 cm, women: >80 cm); blood pressure ≥130/85 mm Hg or drug treatment for essential hypertension; serum high-density lipoprotein cholesterol (HDL-C) level <40 mg/dL (1 mmol/L) in men and <50 mg/dL (1.3 mmol/L) in women or drug treatment for low HDL-C; serum triglycerides (TG) level ≥150 mg/dL (1.7 mmol/L) or drug treatment for elevated TG; and fasting plasma glucose level ≥100 mg/dL (5.6 mmol/L) or drug treatment for DM.

### *H Pylori*-Related Extra-Digestive Diseases

*H pylori* infection may lead to the development or exacerbation of some extra-digestive diseases, such as osteoporosis, obesity, autoimmune thyroiditis, lupus, refractory anemia, and idiopathic thrombocytopenia.^31–33^ Since these extra-digestive diseases may affect MS,^31,34^ individuals with *H pylori*-related or exacerbated extra-digestive diseases were excluded from this study. The FRAX calculation tool and international osteoporosis foundation (IOF) 1-minute risk test were used to screen for the patients with a high risk of osteoporosis. Subjects who had both *H pylori* infection and a high risk of osteoporosis but did not take osteoporosis medications or steroids, did not have underlying diseases related to osteoporosis, such as rheumatoid arthritis and thyroid or parathyroid disorder, did not undergo hormone replacement therapy after oophorectomy before the age of 50 years, and were not alcohol abusers or chain smokers were excluded from this study.

The World Health Organization obesity criterion for Asians (body mass index ≥27.5 kg/m^2^) was used. Patients with *H pylori* infection and obesity who did not have systemic diseases related to obesity, such as DM and endocrine disorder, and were not taking steroids or undergoing hormone therapy were excluded.

Hematological diseases (refractory anemia and idiopathic thrombocytopenia) and autoimmune diseases (autoimmune thyroiditis, lupus, and rheumatoid arthritis) were detected based on the results of laboratory tests and the initial questionnaire.

### Statistical Analysis

For continuous variables, values are expressed as means ± standard deviations (SD). Categorical data were analyzed with the Chi-square test or Fisher exact test, as appropriate. All statistical tests were 2-tailed. A *P-*value of <0.05 was considered to indicate a statistically significant difference. Correlation coefficients such as Pearson correlation coefficient, Point bi-serial correlation coefficient, and Spearman rho were chosen based on data types, including numerical, nominal, and ordinal data. Spearman rho was utilized for ordinal data, such as vitamin D level. The associations among factors such as metabolic parameters, *H pylori* infection (UBT), and vitamin D level were evaluated using the Pearson or Spearman correlation coefficient and multivariate logistic regression analysis after adjusting for potential confounders, such as age or sex.

Receiver operating characteristics (ROC) analysis with maximization of Youden index (sensitivity + specificity − 1) was employed to establish the optimal cut-off level of serum vitamin D predicting MS.

Statistical analyses were performed using SPSS for Windows (Version 16.0, SPSS Inc., Chicago, IL).

## RESULTS

**FIGURE 1:**
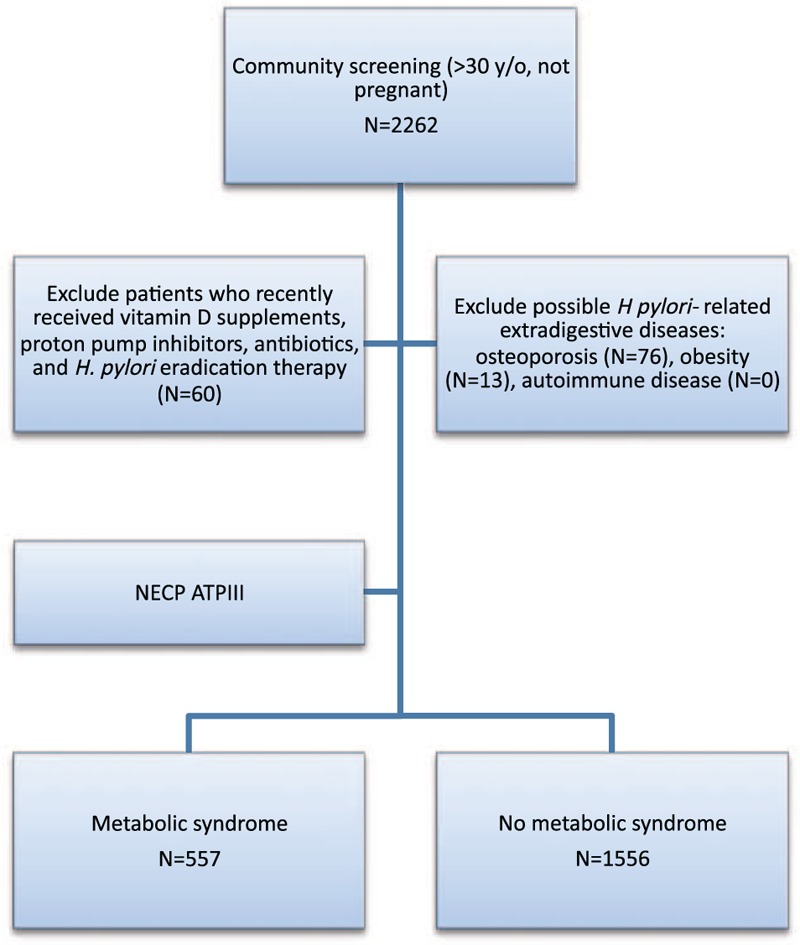
Flow diagram.

A total of 2113 individuals (792 men and 1321 women) were included in this study (Figure 1, flow chart). The demographic data are shown in Table 1. The mean age was 56.4 ± 13.0 years. Based on the ATP III criteria, a total of 557 patients (26.3%) had MS. The participants were divided into 2 groups according to MS status. UBT-positivity (*H pylori* infection) was detected in 53.3% (1126/2113) of the individuals. Serum vitamin D deficiency (<20 ng/mL) was found in 19.8% (419/2113) of the individuals.

**TABLE 1:**
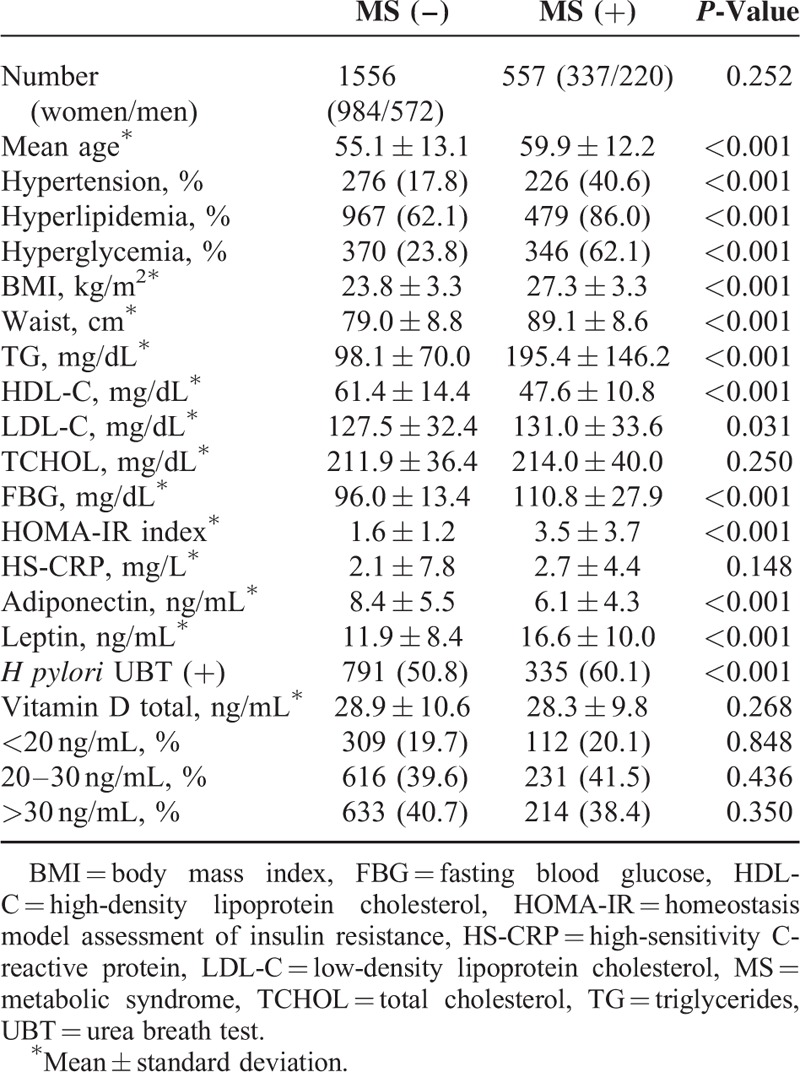
Demographic, Metabolic, and Anthropometric Characteristics of Individuals With and Without Metabolic Syndrome

Individuals with MS were older and more likely to have hypertension, hyperlipidemia, hyperglycemia, and higher mean HOMA-IR scores than those without MS. Participants with MS also had a higher rate of *H pylori* infection than those without MS (60.1% vs 50.8%, *P* < 0.001). However, there were no statistically significant differences in serum vitamin D concentration or vitamin D status (level) distribution between these 2 groups (Table 1).

**FIGURE 2:**
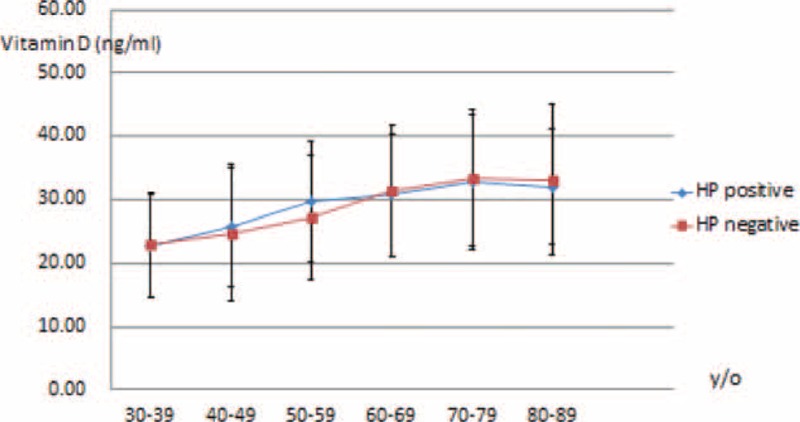
Vitamin D values by age stratification in participants with or without *Helicobacter pylori* infection.

Correlations between presence of MS and *H pylori* infection, vitamin D level, and other factors (adiponectin, leptin, and HS-CRP concentrations) are shown in Table 2. Vitamin D level was positively correlated with age, sex, and adiponectin level but negatively correlated with leptin level. However, presence of *H pylori* infection was not correlated with levels of adiponectin, leptin, or HS-CRP. As both vitamin D level and presence of *H pylori* infection were correlated with age, a further analysis with age stratification was performed to compare mean serum vitamin D levels. No significant difference in mean serum vitamin D level was found between individuals with and without *H pylori* infection (Figure 2).

**TABLE 2:**
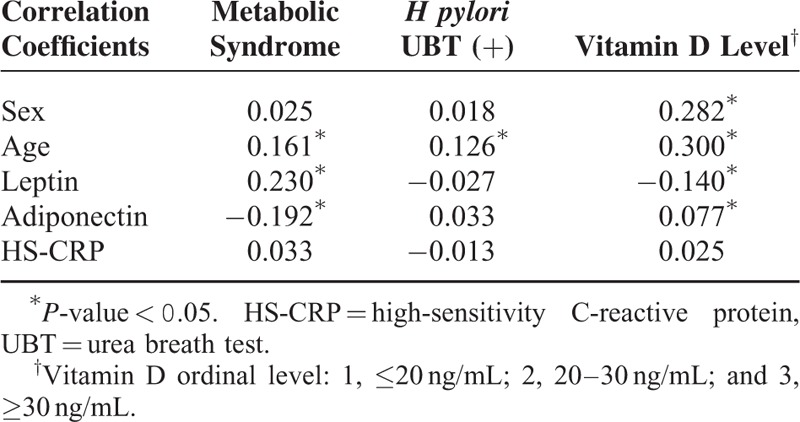
Correlations Between Metabolic Syndrome, *H pylori* Status, Serum Vitamin D Level, and Other Factors

Our attempt to determine the optimal cut-off level of serum vitamin D predicting MS by maximization of Youden index and ROC curves analysis was unsuccessful.

According to logistic regression analysis with adjustments for age and sex, the estimated odds for MS development in individuals with positive UBT compared to those with negative UBT were 1.503 (95% CI: 1.206–1.872, *P* < 0.001) (Table 3). After adjusting for age and sex, individuals with serum vitamin D deficiency (<20 ng/mL) had the odds of MS development of 1.423 (95% CI: 1.029–1.967, *P* = 0.033) compared to subjects with sufficient vitamin D levels (>30 ng/mL) (Table 4).

**TABLE 3:**
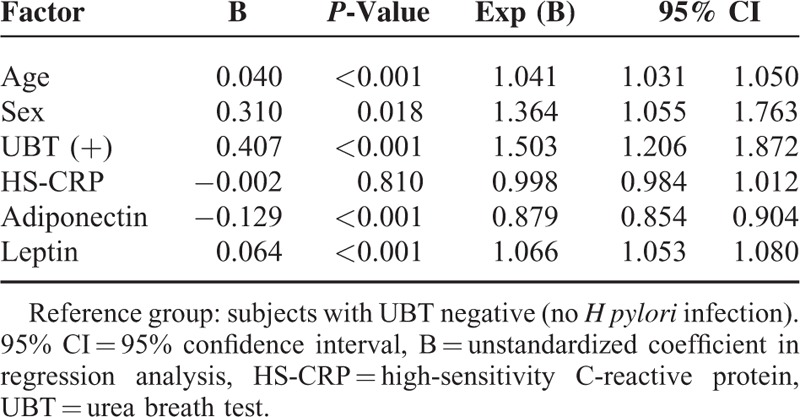
Logistic Regression Analysis of Age, Sex, HS-CRP, Adiponectin, Leptin, and *H pylori* UBT Status as Predictors of Metabolic Syndrome

**TABLE 4:**
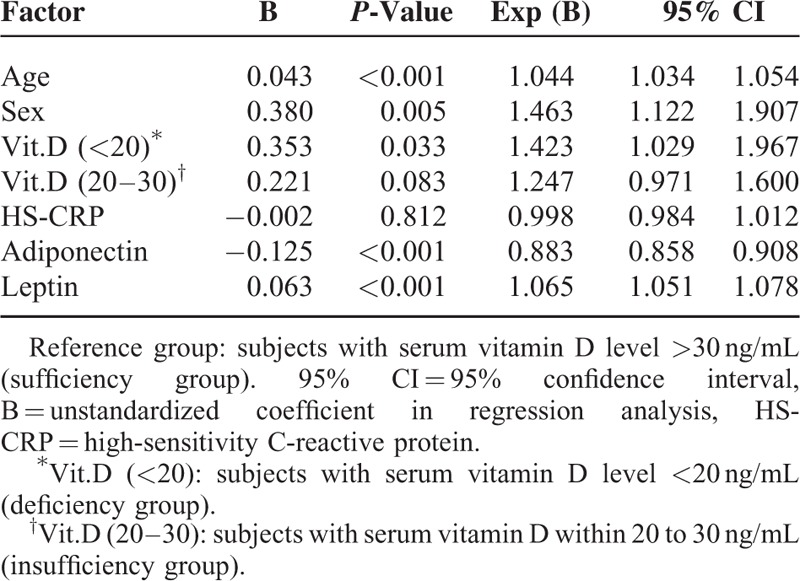
Logistic Regression Analysis of Age, Sex, and Vitamin D Status as Predictors of Metabolic Syndrome

Table 5 presents the results of logistical regression analysis of MS risk depending on age, sex, and serum vitamin D level combined with *H pylori* infection status (positive or negative UBT). Patients with *H pylori* infection and vitamin D deficiency (<20 ng/mL) had the odds of MS development of 2.140 (95% CI: 1.348–3.398, *P* = 0.001) compared to subjects without *H pylori* infection and with sufficient serum vitamin D levels (>30 ng/mL).

**TABLE 5:**
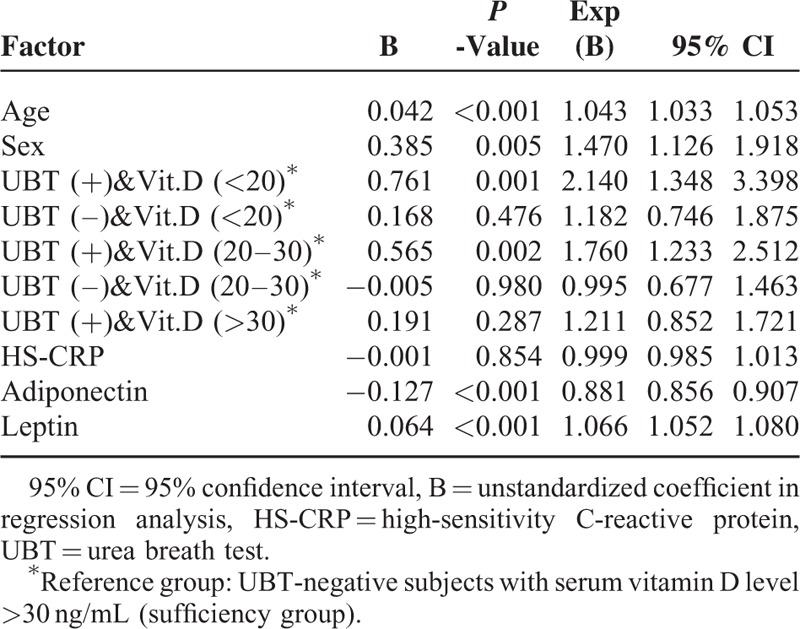
Logistic Regression Analysis of Age, Sex, and a Combination of *H pylori* Infection and Vitamin D Status as Risk Factors of Metabolic Syndrome

A further subgroup analysis performed to compare the distribution of the traits of the MS criteria revealed that individuals with vitamin D deficiency (<20 ng/mL) and *H pylori* infection had a higher rate of abnormal HDL-C level (men: <40 mg/dL, women: <50 mg/dL) than those with sufficient vitamin D levels (>30 ng/mL) and no *H pylori* infection (32.4% vs 15.6%, *P* < 0.001). There were no significant differences in the remaining traits, including waist circumference, fasting sugar level, hypertension, and TG level, between these 2 groups.

## DISCUSSION

The hypothesis of this study was that *H pylori* infection and vitamin D deficiency induce inflammatory cytokines (HS-CRP) or adipokines (adiponectin, leptin), leading to the development of MS. The correlation analysis revealed that vitamin D deficiency was associated with decreased adiponectin level but also with increased leptin level, a combination that has been linked to HDL-C abnormality.^35,36^ Abnormally low HDL-C level is one of the criteria used to diagnose MS, which may explain the association of vitamin D deficiency with MS. However, *H pylori* infection was not correlated with adiponectin, leptin, or HS-CRP level. We surmise that the link between *H pylori* infection and MS status may involve other adipokines or inflammatory cytokines.

*H pylori* infection and serum vitamin D are linked via gastric vitamin D receptor and systemic immune response to chronic gastritis.^20–22^ Thus, a previous study conducted in Italy demonstrated that patients with *H pylori*-related gastritis had lower serum vitamin D levels.^20^ However, there was no statistical difference in mean vitamin D level between individuals with and without *H pylori* after age stratification analysis in the present study. As endoscopic examination was not performed for all the subjects in the present study, it remains unclear whether only patients with *H pylori* infection-related gastritis had lower vitamin D levels.

Several studies have addressed the effects of *H pylori* infection on IR and MS. According to Gunji et al,^37,38^ who conducted 2 large population studies of middle-aged (less than 50 years old) individuals in Japan, *H pylori* infection was significantly associated with MS and IR. Our previous study revealed that the association between *H pylori* infection and MS was stronger in individuals aged less than 50 years.^39^ A recent Taiwanese study of employer checkup data of middle-aged individuals, which also employed the ^13^C UBT survey revealed that *H pylori* infection was positively associated with MS, especially in women.^40^ The current study also showed that, after adjusting for age and sex, *H pylori* infection was a predictor of MS development.

Although some past studies revealed that vitamin D deficiency was common in patients with MS, the relationship between the 2 depends on the geographical area (Table 6), which may be partially explained by differences in exposure to ultraviolet B (UVB) radiation. UVB exposure varies greatly by residence latitude, season, and degree of skin pigmentation.^44^ Above the 37° N latitude, skin vitamin D production decreases during winter (November to February) because of reduced UVB exposure.^45,46^ According to a meta-analysis, most studies that confirmed the association of blood vitamin D level with the risk of MS were cross-sectional studies conducted in regions north of 38° N.^18^ In contrast, most studies that found no such association were from lower latitude areas, with more sunshine and higher temperatures. In agreement, the present study, which was conducted in Keelung City (latitude: 25° N), did not find statistically significant differences in mean serum vitamin D level and distribution of individuals in vitamin D status groups between participants with and without MS. As vitamin D level was positively correlated with age and sex, a regression analysis of the association between vitamin D level and MS was performed after adjusting for these factors. This analysis yielded an odds ratio of 1.450 for MS development in patients with vitamin D deficiency (<20 ng/mL) compared to subjects with sufficient vitamin D levels (>30 ng/mL). Moreover, this odds ratio increased to 2.140 for patients with *H pylori* infection as determined by the UBT and vitamin D deficiency (<20 ng/mL) when compared to UBT-negative participants with sufficient vitamin D levels (>30 ng/mL).

**TABLE 6:**
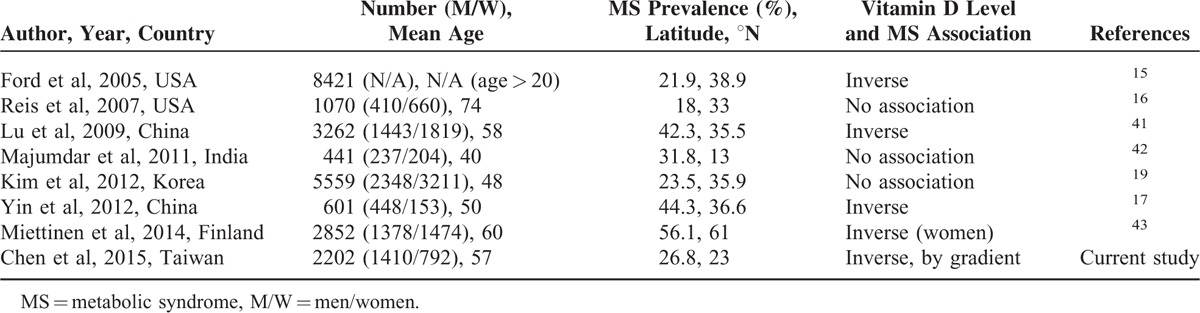
Recent Studies of the Associations Between Vitamin D and Metabolic Syndrome

There were some limitations to this study. First, because the study was based on community-based health screening data, selection bias cannot be excluded. Moreover, considering that our participants were relatively old (56.4 years) and that the investigation was conducted in a single location (25° N) characterized by a high *H pylori* infection prevalence (53.3%), the results cannot be generalized to all populations and geographical regions. Second, esophagogastroduodenoscopy was not performed for all participants with *H pylori* infection. There was no information about gastritis, malignancy, *H pylori* virulence, and stomach vitamin D receptor status. Nevertheless, the study revealed a possible link between vitamin D level and MS mediated by adiponectin, leptin, and HDL. This warrants further investigation of the association between vitamin D, adipokines, lipids, and MS. Other cytokines or adipokines may be investigated for their involvement in the pathogenic mechanisms of *H pylori* and MS.

In conclusion, *H pylori* infection could be a predictor of MS. Individuals with vitamin D deficiency are at a higher risk of MS than those with normal vitamin D levels. Finally, the odds ratio for MS development in patients with both *H pylori* infection and vitamin D deficiency is 2.140 when compared to individuals without *H pylori* infection and with sufficient vitamin D levels.
